# Les tumeurs neuroendocrines du col utérin: à propos d’un cas avec revue de la littérature

**DOI:** 10.11604/pamj.2019.34.48.19861

**Published:** 2019-09-24

**Authors:** Kaoutar Rais, Ebo Egyir, Obeid Rockson, Abdelbassir Ramdani, Badr Serji, Tijani El Harroudi

**Affiliations:** 1 Service de Chirurgie Oncologique, Centre d’Oncologie Hassan II, Oujda, Maroc

**Keywords:** Carcinome neuroendocrine, col utérin, colpohystérectomie, radiothérapie, chimiothérapie, Neuroendocrine tumor, cervix, colpohysterectomy, radiotherapy, chemotherapy

## Abstract

Les carcinomes neuroendocrines du col utérin sont des carcinomes rares qui ne représentent que 0,9-1,5% des tumeurs du col. Le diagnostic est établi tardivement vu la non spécificité des signes cliniques et l'absence d'un test de dépistage. Les études immunohistochimique et histologique jouent un rôle crucial pour confirmer ce type de tumeurs. A nos jours, leurs prise en charge thérapeutique est difficile et leurs pronostic reste toujours défavorable. Nous rapportons un cas de carcinome neuroendocrine du col utérin et nous discuterons les particularités de cette entité rare.

## Abstract (en)

Neuroendocrine tumors of the cervix are rare accounting for only 0.9-1.5% of these tumors. The non-specificity of the clinical signs and the absence of a screening test delay diagnosis. Immunohistochemical and histological studies play a crucial role to confirm the diagnosis. Today, therapeutic management is difficult and prognosis is unfavorable. We report a case of neuroendocrine tumor of the cervix and we will outline the peculiarities of this rare disease.

## Introduction

Les tumeurs neuroendocrines du col utérin représentent 0,9 à 1,5% des tumeurs cervicales [1]. Ce sont des tumeurs rares, agressives et de mauvais pronostic dont la prise en charge thérapeutique demeure à nos jours un dilemme entre plusieurs équipes médicales. Nous rapportons un cas d'une tumeur neuroendocrine du col utérin durant l'année 2018 au centre d'oncologie Hassan II Oujda.

## Patient et observation

**Figure 1:**
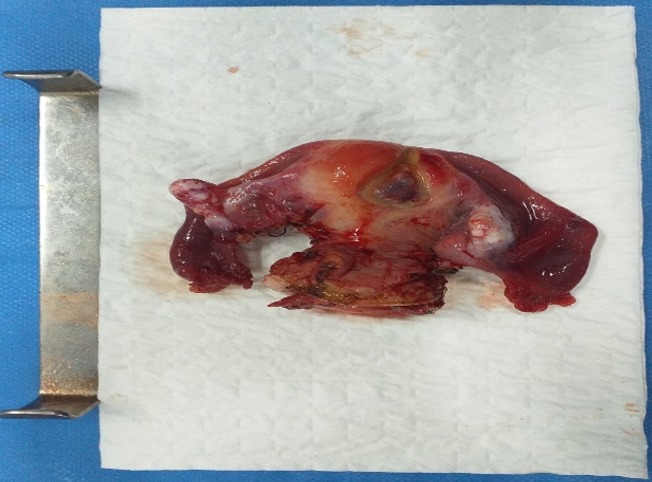
Image montrant la pièce opératoire de la colphystérèctomie élargie

**Figure 2:**
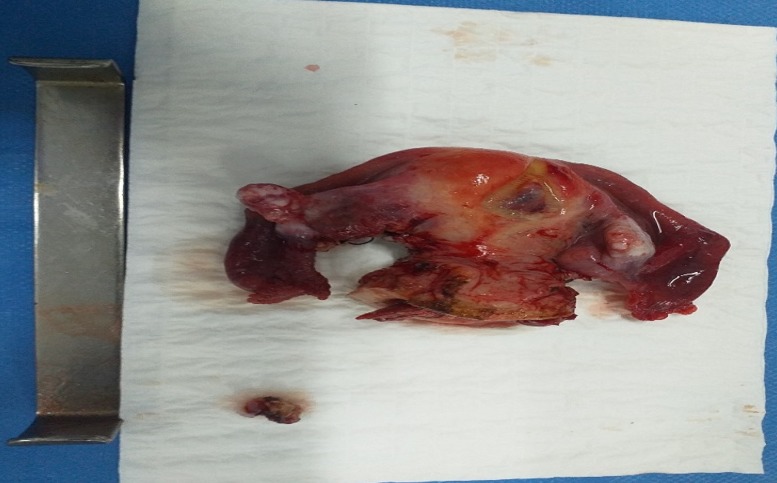
Image montrant la pièce opératoire de la colphystérèctomie élargie et de la recoupe vaginale

Il s'agit d'une patiente âgée de 54 ans, mariée, G6P5, sans aucun autre antécédent ni personnel ni familial. Elle a consulté pour des métrorragies intermittentes de faible abondance évoluant depuis 5 mois avant son admission, sans signes digestifs ni urinaires ni autres signes gynécologiques associés. Le tout évoluant dans un contexte de conservation de l'état général. Aux touchers pelviens, on note la présence d'un processus de 6 cm au niveau du col utérin, infiltrant les culs de sacs vaginaux qui arrive jusqu'à la jonction 1/3 moyen et 1/3 inférieur du vagin, les paramètres droit et gauche étaient libres. Des biopsies réalisées à ce niveau, sont revenues en faveur d'une prolifération de cellules carcinomateuses agencées en plages compactes, ébauchant des formations tubulo-glandulaires, adossées les unes aux autres réalisant par place un aspect cribriforme. Les atypies cyto-nucléaires étaient modérées, les mitoses peu nombreuses et le stroma était inflammatoire. Un complément par une étude immuno-histochimique des différents fragments a objectivé un marquage des cellules carcinomateuses à l'aide des anticorps: anti-synaptophysine, anti-NSE (Neuron Specific Enolase) et l'anticorps anti-chromogranine. Ceci rend cette tumeur compatible à un carcinome neuroendocrine peu différencié invasif du col utérin. Un scanner thoraco-abdomino-pelvien a montré la présence d'une tumeur au niveau du col utérin classée IIIB avec un utérus augmenté de taille, de contours bosselés refoulant la vessie en avant, avec persistance de liseré de séparation graisseux et exerçant une empreinte sur le rectum, avec une absence d'adénopathies profondes et d'autres signes d'extension de la pathologie tumorale. La décision retenue en réunion de concertation pluridisciplinaire était une radio-chimiothérapie concomitante. La patiente a bénéficié de 5 cures de chimiothérapie à 1 semaine d'intervalle à base de CDDP (le cisplatine ou cis-diaminedichloroplatine) 68mg en concomitant avec la radiothérapie à la dose 46 Gray sur le pelvis en 30 séances, 10 Gray sur les paramètres et 4 Gray sur les adénopathies. L'évaluation clinique post-thérapeutique a montré la persistance d'une tumeur mesurant presque 4cm infiltrant toujours les culs de sacs vaginaux d'où l'indication d'un traitement chirurgical qui a consisté en une colpohystérerctomie élargie de clôture (Figure 1, Figure 2) avec des suites post-opératoires simples. Le résultat anatomopathologique a montré l'absence de territoire suspect de malignité sur la pièce opératoire ce qui prouve la bonne réponse thérapeutique au traitement néoadjuvant. La patiente a évolué favorablement durant les 4 mois qui suivent la fin du traitement.

## Discussion

Les tumeurs neuroendocrines se développent principalement au niveau du tractus digestif et les poumons; au niveau du col utérin, elles ne représentent que 0,9 à 1,5% des tumeurs du col utérin qui sont généralement prédominées par le carcinome épidermoïde [2]. Elles se manifestent cliniquement par des méno-métrorragies et des leucorrhées, exceptionnellement par un syndrome paranéoplasique (syndrome de Cushing, syndrome carcinoïde, hypoglycémie, syndrome de sécrétion inappropriée d'hormone anti-diurétique, hypercalcémie) [3]. Le diagnostic se base essentiellement sur les données histologiques et l'étude immunohistochimique. On distingue quatre types de carcinome neuroendocrine: les tumeurs carcinoïde typique, atypique, carcinome à petites cellules et carcinomes à grandes cellules dites de haut grade. Les cellules endocrines possèdent une diversité considérable de taille, d'argyrophilie, de colorations immunohistochimiques et de l'ultrastructure. Elles peuvent être identifiées en histochimie grâce à la coloration de Grimelius par la mise en évidence de granulations argyrophiles ou neurosécretoires, en immunohistochimie par une positivité à la NSE, la chromogranine, la synaptophysine et aux anticorps pour la gastrine, l'insuline ou par la production ectopique de l'hormone corticotrope (ACTH), de ßMSH, sérotonine, d'histamine et d'amylose [4]. Les carcinomes neuroendocrines à petites cellules sont les tumeurs ayant le plus mauvais pronostique. Ils présentent des similitudes avec les carcinomes à petites cellules du poumon, vu qu'elles sont dotées d'un index mitotique élevé, une nécrose étendue, et une invasion lymphatique et vasculaire massive [5]. Ils se distinguent des carcinomes épidermoïdes par leurs taux de récidive plus élevé et le retard de leur diagnostic à cause de l'inefficacité de dépistage de ce type de tumeurs par le frottis cervico-utérin. En revanche, l'association avec le “human papillomavirus (HPV)” 16 et 18 constitue un facteur de risque en commun entre les deux carcinomes. D'ailleurs, selon une étude réalisée en 2018 concernant la contribution de l'HPV sur la formation des tumeurs neuroendocrines du col à propos d'une série de 10575 cas de tumeurs invasives du col; HPV DNA a été détecté dans 85,7% des cas de tumeur neuroendocrine (HPV16 54.8% et HPV18 40.5%) [6].

**Figure 3:**
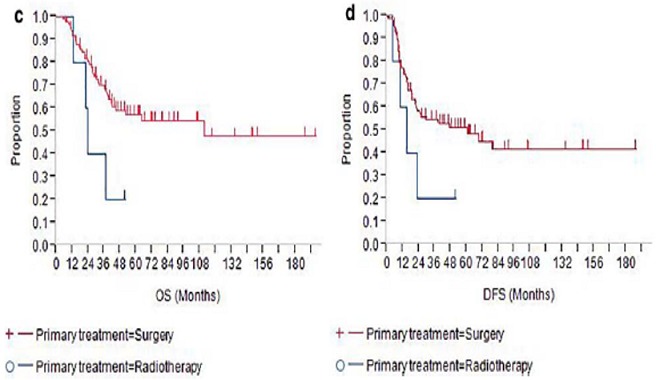
Comparaison du taux de survie et la survie sans maladie entre deux populations l’une traitée initialement par la chirurgie et l’autre par la radiothérapie

Le traitement des carcinomes neuroendocrines du col utérin repose sur la chirurgie, la chimiothérapie ou la radiothérapie. La décision thérapeutique dépend essentiellement du stade FIGO, la taille tumorale, le staging ganglionnaire et la présence ou non de métastases à distance [7]. Selon une étude récente publiée en 2017 sur les facteurs pronostiques et le traitement optimal des tumeurs neuroendocrines du col classées I et II à propos de 198 cas au service gynéco-obstétrique du Japon: 88 patientes (94.6%) (Tableau 1) ont reçu une hystérectomie radicale avec un curage pelvien, 18 parmi elles ont bénéficié d'une chimiothérapie néoadjuvante, 14 patientes ont reçu une radiothérapie adjuvante, 48 une chimiothérapie adjuvante et 11 patientes une radio-chimiothérapie adjuvante [8]. Ils ont conclu que la chirurgie radicale en première intention est plus efficace que la radiothérapie seule avec un taux de récidive locorégionale moins élevé. La chimiothérapie adjuvante à base de EP (etoposide + platinum)/CPT-P (irinotecan + platinum) avait de très bon résultat dans cette série avec un taux de survie élevé en comparaison avec une radiothérapie adjuvante (Figure 3). Par contre, deux auteurs ont rapporté des résultats décevants concernant le traitement local (chirurgie avec ou sans radiothérapie) des tumeurs classées stade I-IIA: Sheet *et al.* [9], les premiers auteurs, ont trouvé un taux de survie globale à trois ans de 16% et un taux de survie sans progression à cinq ans de 0%. Pour Sevin *et al.* [10], le taux de survie sans maladie à 5 ans était de 36%. Des rechutes principalement hématogènes (67 à 90% des cas) et ganglionnaires (34% des cas), une incidence élevée d'adénopathies au diagnostic (40-60%), et une invasion vasculaire fréquente, sont autant de facteurs qui ont incité la majorité des auteurs à associer un traitement systémique au traitement local [11].

**Tableau 1:** Traitement initial reçu par les patientes de la série Japonaise

Traitement reçu	Nombre de patients		Pourcentage%
Chirurgie radicale	Totale	88	94,6
	Chirurgie seule	14	15,1
	CMTNA+chirurgie	1	1,1
	CMTNA+chirurgie+RTHA/RCC	2	2,2
	CMTNA+chirurgie+CMTA	11	11,8
	CMTNA+chirurgie+RTHA/RCC+ CMTA	4	4,3
	chirurgie+RTHA/RCC	12	12,9
	chirurgie+CMTA	37	39,8
	chirurgie+RTHA/RCC+ CMTA	7	7,5
Radiothérapie définitive	Totale	5	5,4
	Radiothérapie	2	2,2
	CMTNA+RCC+CMTA	1	1,1
	RCC+CMTNA	2	2,2

**Abréviations:** CMTNA=Chimiothérapie néoadjuvante, RTHA=radiothérapie adjuvante

RCC=Radiochimiothérapie concomitante, CMTA=Chimiothérapie adjuvante

**Figure 4:**
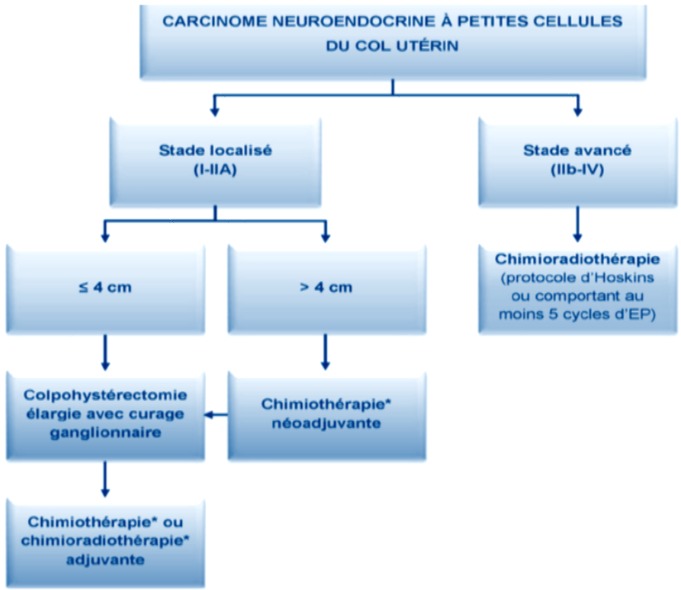
Les recommandations de la prise en charge thérapeutique des carcinomes neuroendocrine du col utérin [18]

Zivanovic *et al.* ont comparé rétrospectivement un traitement local seul (chirurgie) et un traitement local associé à une chimiothérapie adjuvante. Ils ont trouvé un taux de survie sans récidive à trois ans de 83% pour les patientes ayant reçu une chimiothérapie à base de cisplatine et d'étoposide contre 0% en cas de traitement local seul [12]. Du fait du taux de dissémination métastatique précoce, certains auteurs ont préféré utiliser une chimiothérapie néoadjuvante; Chang a démontré une réponse complète de 6 sur 7 patientes ayant reçu le VAC/PE avant l'hystérectomie[13]. Par contre, Lee *et al.* n'ont pas objectivé de bénéfice sur les 11 patientes ayant reçu une thérapie néoadjuvante (2 patientes sur 5 qui avaient des TNE classées IB1 et les 6 autres patientes avec des tumeurs IB2-IIA ayant bénéficié d'une chimiothérapie néoadjuvante sont décédé dans les 2 années suivantes) [14]. Pour les tumeurs localement évoluées (stades IIb-IV) et pour les patientes inopérables, une association de radiothérapie et de chimiothérapie est préconisée, selon le protocole d'Hoskins *et al*. [15]. A ces stades, une chimiothérapie comportant au moins cinq cures de cisplatine et d'étoposide est associée à une meilleure probabilité de survie sans récidive. D'ailleurs c'était le protocole utilisé chez notre patiente avec une nette amélioration clinique après la chimiothérapie néoajuvante associée à la radiothérapie. Pour les tumeurs à un stade avancé, elles sont traitées par une chimiothérapie combinée à base de platines. En cas de récurrence ou de chimiorésistance, une thérapie 2^ème^ ligne est mise en route par vincristine/doxorubicin/cyclophosphamide et topotecan [16]. En cas de maladie métastatique ou de récidive, une chimiothérapie, comportant soit du cisplatine et de l'étoposide seuls, soit en alternance avec une chimiothérapie de type VAC (vincristine, adriamycine et cyclophosphamide) est indiquée [17]. En général, selon les recommandations publiées par Chan *et al.* en 2003 [18], le traitement chirurgical est indiqué essentiellement pour les TNE classées I-IIA, mesurant moins de 4cm suivi parfois d'une chimiothérapie ou radio-chimiothérapie adjuvante, alors que les tumeurs I-IIA mesurant plus de 4cm une chimiothérapie néoadjuvante est préconisée avant la chirurgie. Pour les tumeurs neuroendocrines du col utérin classées IIb-IV, une radiochimiothéparpie en appliquant le protocole d'Hoskins est souhaitable (Figure 4).

## Conclusion

Les tumeurs neuroendocrines du col utérin sont des tumeurs très agressives et rares ce qui explique l'absence d'essais randomisés et ce qui rend leur prise en charge de plus en plus difficile.

## Conflits d’intérêts

Les auteurs ne déclarent aucun conflit d'intérêts.
